# Translation and validation of the TEAM-NL instrument for assessing interprofessional team performance in simulation-based resuscitation training

**DOI:** 10.1016/j.resplu.2026.101535

**Published:** 2026-10-20

**Authors:** Frederik Marynen, Lars Mommers, Dennie Wulterkens, Thom Bormans, Bas C.T. van Bussel, Iwan C.C. van der Horst, Daniëlle Verstegen, Diana Dolmans, Walther N.K.A. van Mook, Simon Cooper

**Affiliations:** a Department of Anaesthesiology, Universitair Ziekenhuis Brussel, Brussels, Belgium; b School of Health Professions Education (SHE), Maastricht University, Maastricht, the Netherlands; c Department of Anaesthesiology and Pain Medicine, Maastricht University Medical Centre, Maastricht, the Netherlands; d Department of Simulation in Healthcare, Maastricht University Medical Centre, Maastricht, the Netherlands; e Quality and Training Time, Houten, the Netherlands; f Department of Intensive Care Medicine, Maastricht University Medical Centre, Maastricht, the Netherlands; g Cardiovascular Research Institute Maastricht (CARIM), Maastricht University, Maastricht, the Netherlands; h Care and Public Health Research Institute (Caphri), Maastricht University, Maastricht, the Netherlands; i Board of Directors, Albert Schweitzer Hospital, Dordrecht, the Netherlands; j Department of Educational Development and Research, Faculty of Health, Medicine and Life Sciences, Maastricht University, Maastricht, the Netherlands; k Academy for Postgraduate Medical Training, Maastricht University Medical Centre, Maastricht, the Netherlands; l Cooper Consultancy, Cowes, Victoria, Australia

**Keywords:** Team performance, Non-technical skills, Resuscitation, Simulation-based training, Team Emergency Assessment Measure

## Abstract

**Background:**

Effective interprofessional teamwork is essential for successful resuscitation care. The Team Emergency Assessment Measure (TEAM) is widely used to assess emergency team performance; however, a validated Dutch version is lacking. This study aimed to translate and validate a Dutch version of the TEAM (TEAM-NL) for simulation-based resuscitation training.

**Methods:**

A structured cross-cultural adaptation was performed, including forward–backward translation, developer review and expert review. Instructors used the TEAM-NL during Advanced Life Support courses at two Dutch training centres, rating each participant group in two simulation scenarios. Internal consistency, inter-rater reliability, convergent validity, change in scores between scenarios, acceptability and score distribution were assessed.

**Results:**

A total of 52 participant groups were observed, yielding 104 observed team performances and 299 instructor ratings. The mean total score (range 0–44) was 32.1 (SD 6.9). Internal consistency was high (Cronbach’s *α* = 0.93). Inter-rater reliability was moderate for two raters (ICC(1,2) = 0.68, 95% CI 0.53–0.79) and good for three raters (ICC(1,3) = 0.78, 95% CI 0.69–0.85). The total score correlated strongly with the global performance rating (*r* = 0.86, *p* < 0.001). Scores were higher in the second scenario (mean difference 4.3, 95% CI 2.2–6.4; *d_z_* = 0.57). Missing data were minimal (0–2.7% per item). No floor effects were observed; ceiling effects were present for all items (19.4–36.1%).

**Conclusion:**

The TEAM-NL showed high internal consistency, convergent validity and acceptability for instructor-based assessment of team performance during simulation-based resuscitation training. Inter-rater reliability was moderate for two raters and good when the ratings of three instructors were averaged.

## Highlights

• TEAM-NL is a translated and psychometrically validated tool to assess team performance in resuscitation settings.
• TEAM-NL demonstrated high internal consistency, moderate-to-good inter-rater reliability and evidence of construct validity.
• TEAM-NL supports standardized assessment during simulation-based resuscitation training.

## Background

The delivery of high-quality healthcare depends on effective interprofessional collaboration. Increasingly complex patient needs, growing specialization of professional roles and mounting time pressures mean that care is seldom delivered by a single professional working in isolation.^1^ Technical competence alone is therefore insufficient, particularly as adverse events are frequently attributable to failures in communication and coordination within teams.^2^, ^3^, ^4^ Consequently, non-technical skills such as leadership and teamwork, in combination with technical skills, are now widely recognized as fundamental to safe and reliable patient care.^5^, ^6^

Interprofessional education aims to prepare healthcare professionals for such collaboration across professional boundaries.^7^ Simulation-based education has become a prominent modality for this purpose, as it allows learners from different professions to engage in realistic scenarios without risk to patients.^8^, ^9^ Team training interventions have been associated with improved team performance^10^, ^11^, ^12^, ^13^ and patient outcomes.^14^

To support meaningful learning, several observational instruments have been developed over the past decades to measure teamwork and other non-technical skills in healthcare simulation and clinical settings.^15^, ^16^, ^17^ These tools typically capture behavioural markers of effective teamwork, enabling educators to provide targeted feedback and to track progress over time.^5^, ^18^ Their usefulness depends on their psychometric quality and their suitability for the context in which they are used.^19^

The Team Emergency Assessment Measure (TEAM) is a widely used instrument for assessing team performance in emergency and acute care settings.^20^ Its brevity, behavioural focus and content validity make it attractive for educational use, including in time-constrained simulation settings.^17^ Evidence for its validity and reliability has been reported in various settings,^20^, ^21^ and translated versions have been evaluated in French,^22^ German,^23^ Swedish,^24^ Brazilian-Portuguese^25^ and Spanish.^26^ However, a validated Dutch version of the TEAM is lacking. This study therefore aimed to translate and validate a Dutch version of the TEAM (TEAM-NL) for use in simulation-based interprofessional resuscitation training, by examining its internal consistency, inter-rater reliability, validity and acceptability when used by ALS instructors.

## Methods

This study employed a structured five-stage approach based on the recommended guidelines for the cross-cultural translation and validation of psychometric instruments.^27^, ^28^ The process consisted of forward translation (Stage 1) and backward translation (Stage 2), followed by review and approval by the original instrument developer (Stage 3). Subsequently, expert validation was conducted (Stage 4), and the final stage involved psychometric validation for instructors’ assessment (Stage 5). An overview of the study design is presented in Fig. 1.

**Fig. 1:**
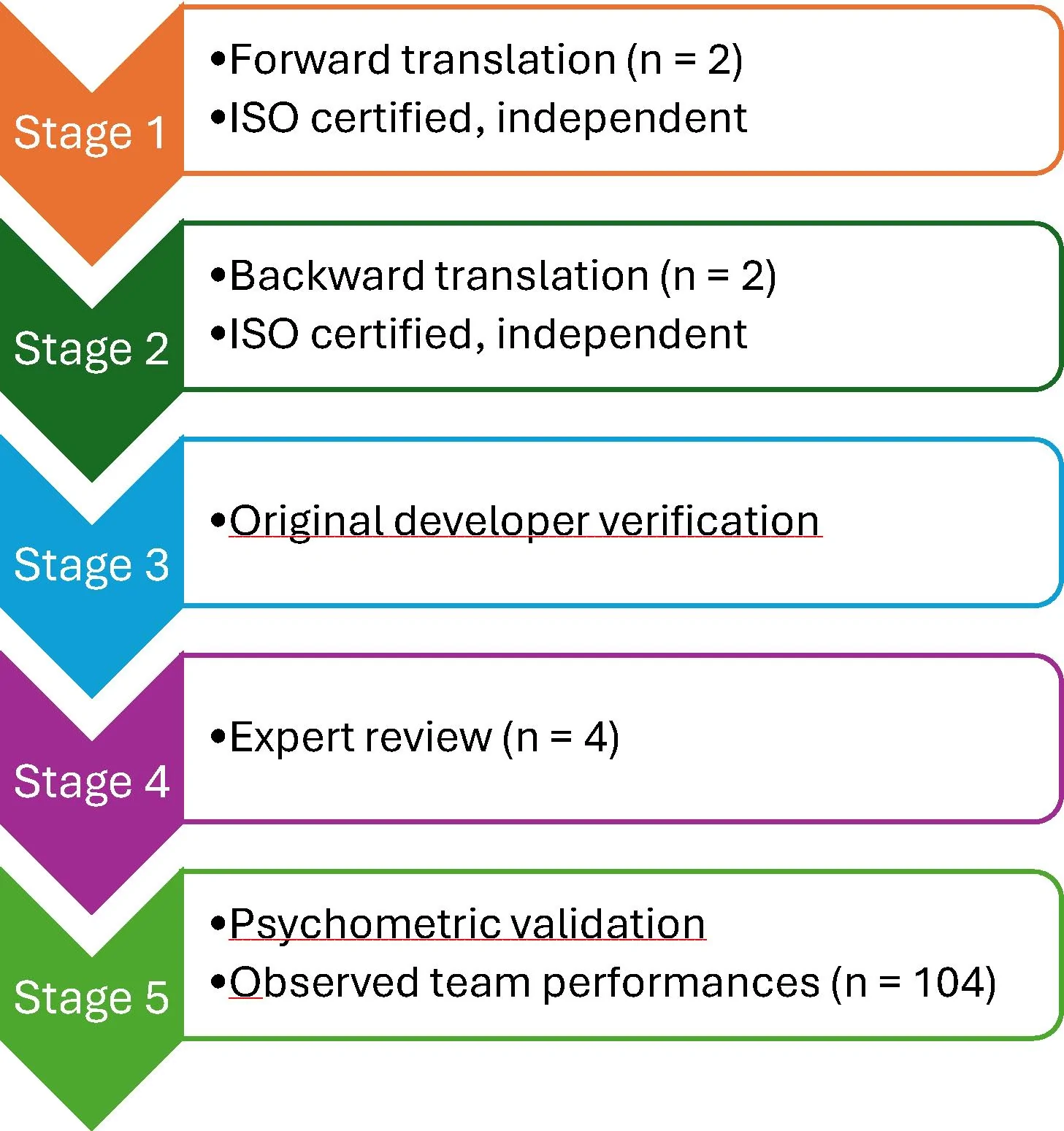
**Stages in the cross-cultural translation and validation of the TEAM-NL instrument**.

### Stage 1 and 2

The original English TEAM instrument was translated into Dutch by two independent ISO 17100:2015-certified medical translators. These forward translations were synthesized into a single reconciled Dutch version through discussion until consensus was reached. This version was reviewed by two content experts (LM, DW) to ensure conceptual and clinical accuracy.

Subsequently, the reconciled Dutch version was back-translated into English by two other independent ISO 17100:2015-certified translators who were blinded to the original instrument. In total, four independent translators were involved across the forward and backward translation stages.

### Stage 3

The synthesized back-translated English version was reviewed and verified by the original instrument developer (SC). The feedback focused on semantic and conceptual equivalence between the original and translated versions. Minor textual revisions were suggested for five elements of the instrument to improve clarity and alignment with the original intent. These revisions were incorporated into the pre-final Dutch version (Supplement 1).

### Stage 4

Content validity of the pre-final Dutch version was reviewed by an expert panel, consisting of two medical doctors and advanced life support instructors (LM, FM), one registered nurse and crisis resource management specialist (Th.W), and one educational specialist (WvM). The panel reviewed the instrument for clarity, relevance, and clinical applicability. Based on their feedback, small textual changes were made to the prompt statements of item 7, which resulted in the approval of the final TEAM-NL instrument (Supplements 2 and 3).

### Stage 5 – Psychometric validation

Psychometric validation was conducted regarding the reliability, validity and acceptability during 19 certified European Resuscitation Council (ERC) ALS (re)certification courses at two training centres in the Netherlands. Each course included two to four groups, consisting of five to six participants. Each group was evaluated during two simulated cardiac arrest scenarios by two different groups of instructors: one at the start of the training and one near the end of the training. Reporting of the reliability analyses was guided by the Guidelines for Reporting Reliability and Agreement Studies (GRRAS).^29^

#### Scoring TEAM instrument

The TEAM tool consists of 11 items scored on a 5-point Likert scale (ranging 0–4) and 1 global rating item (ranging 1–10). The items cover three categories: leadership, teamwork and task management. Relevant prompts are provided to aid rating of the individual items.^20^

#### Application of the Dutch TEAM tool in an advanced life support training environment

Participants were recruited from certified ERC Advanced Life Support (re)certification courses. Participants included medical consultants, resident physicians, and registered nurses from hospitals across the Netherlands. All instructors were certified ERC resuscitation instructors. The instructors received no specific rater training in the use of the TEAM-NL instrument. Participation in the study was voluntary and data were collected anonymously.

#### Scenarios

The resuscitation teams managed standardized simulated resuscitation scenarios in accordance with the course regulations. The first observed scenario involved either ventricular fibrillation or pulseless ventricular tachycardia due to acute coronary occlusion or pulseless electrical activity secondary to pulmonary embolism. The second observed scenario varied according to ALS course specifications and included anaphylaxis, postoperative hypovolaemia, trauma following a motor vehicle accident or severe asthma. All scenarios were conducted in an immersive simulation environment using standardized equipment and structured scenario scripts.

### Ethical considerations

All course participants and instructors received written information about the study at least four weeks prior to the resuscitation course. Participation was voluntary. Participants and instructors provided written informed consent through an explicit opt-in on their respective questionnaire forms, including consent for the use of anonymized data for research purposes. Participants also completed the TEAM-NL as a self-assessment; these data are reported separately. No participant or instructor declined consent. The study was approved by the Ethics Review Committee of the Faculty of Health, Medicine and Life Sciences of Maastricht University (2024-141).

### Sample size calculations

The sample size was determined using the approach of Walter et al.^30^ for reliability studies, assuming a minimum acceptable reliability and a higher expected reliability, with *α* = 0.05 and 80% power. Using a conservative assumption of *k* = 2 raters per target, the required number of targets (observed performances) was *n* = 102 when testing a minimum acceptable ICC of 0.60 against an expected ICC of 0.75. Because reliability was reported for the average of *k* raters per target (ICC(1,*k*)), sample size calculations accounted for the number of raters per target.

A secondary precision-based calculation using the method of Bonett^31^ for estimating ICC precision for a one-way random-effects ICC, assuming an expected ICC of 0.75 and a desired 95% confidence interval (95% CI) half-width of 0.10 with *k* = 2 raters per target, yielded *n* = 75 targets. Therefore, an effective sample size of at least 102 observed performances was deemed sufficient.

### Data collection

Immediately after each resuscitation scenario, all instructors independently completed the TEAM-NL instrument on paper forms, which were collected in sealed envelopes immediately after completion. Each scenario was planned to be assessed by at least two instructors, with an additional third, and occasionally a fourth, instructor when feasible within course logistics. TEAM-NL ratings were not discussed during the course to prevent influence on subsequent scoring. Data collection was conducted between June 2025 and January 2026.

### Statistical analysis

Analyses were conducted at different levels depending on the research question. Internal consistency and convergent validity were evaluated at the individual instructor-rating level, inter-rater reliability at the observed-performance level and change in scores between scenarios at the participant-group level.

Statistical analyses were performed using IBM SPSS Statistics (version 31.0.0.0). Descriptive statistics were calculated for TEAM-NL item scores, subscale scores, and total scores and reported as means with standard deviations (SD). Total scores were calculated as the sum of items 1–11. For forms with one missing item, the total score was prorated using the mean of the available items; forms with more than one missing item (*n* = 3) were excluded from analyses involving the total score. The Teamwork subscale score was prorated when one of its seven items was missing; Leadership and Task Management scores required both items. A complete-case sensitivity analysis was performed.

Regarding reliability, internal consistency was evaluated using Cronbach’s alpha for the overall 11-item TEAM-NL scale and the seven-item Teamwork subscale. Alpha coefficients ≥0.70 were considered acceptable. For the two-item Leadership and Task Management subscales, inter-item correlations and Spearman-Brown coefficients were examined to assess item homogeneity.^32^ Corrected item-total correlations were calculated for all items. All internal consistency analyses were based on forms with complete data for items 1–11 (listwise deletion; *n* = 275). Inter-rater reliability (IRR) was assessed using intraclass correlation coefficients (ICC) based on a one-way random-effects model. Average-measures ICCs with 95% confidence intervals (95% CIs) were reported to reflect the reliability of the mean instructor rating for an observed team performance. ICC(1,2) and ICC(1,3) were calculated using all valid total scores with at least two and three available instructor ratings, respectively. When more ratings were available than required for a given analysis, the first two or three ratings in data-entry order were used; data-entry order was arbitrary and not determined by instructor characteristics. ICC values <0.50, 0.50–0.75, 0.75–0.90 and >0.90 were interpreted as indicating poor, moderate, good, and excellent reliability, respectively.^33^

To account for potential dependency between ratings of the same performance and repeated observations of the same team across scenarios, sensitivity analyses for internal consistency were performed separately for each scenario and rating position. Similarly, sensitivity analyses for inter-rater reliability were performed separately for each scenario to assess the robustness of the overall ICC estimates. Acceptability and score distribution were assessed using missing responses, completion rates, and floor and ceiling effects for each item. Next, convergent validity was assessed using Pearson’s correlation coefficients between the global performance rating (item 12) and both the TEAM-NL total score (items 1–11) and the subscale scores. To allow comparison with the original TEAM validation,^20^ Spearman’s *ρ* was additionally calculated between the total score and global performance rating.

Finally, change in TEAM-NL scores between the first and second simulation scenario was examined at the participant-group level. For each participant group, the mean total score across instructors was calculated per scenario, and the two group means were compared using a paired-samples *t*-test. The normality of the difference scores was checked using Shapiro-Wilk test and *Q*–*Q* plots. Effect sizes were calculated as Cohen’s *d*, based on the standard deviation of the difference scores (*d_z_*). Statistical significance was set at *p* < 0.05.

## Results

A total of 52 participant groups each completed two scenarios, resulting in 104 observed team performances. Because one instructor became unavailable during one course, two performances were rated by only one instructor; all other performances were rated by two to four instructors. The mean number of ratings per observed performance was 2.9 (SD 0.5), resulting in 299 instructor ratings. No instructors declined participation. Three forms (1.0%) had more than one missing item and therefore no valid total score. The mean TEAM-NL total score at the rating level was 32.1 (SD 6.9, *n* = 296) (Table 1).

**Table 1:** Overview of observations and instructor ratings.

**Characteristic**	**Value**
Participant groups, *n*	52
Observed team performances, *n*	104
**Instructor ratings per observed performance, *n* (%)**	
1 rating	2 (1.9)
2 ratings	12 (11.5)
3 ratings	87 (83.7)
4 ratings	3 (2.9)
Mean (SD)	2.9 (0.5)
Instructor ratings, total, *n*	299
Scenario 1, *n*	149
Scenario 2, *n*	150
TEAM-NL total score, mean (SD)*	32.1 (6.9)

* Range 0–44; prorated for forms with one missing item (*n* = 296).

### Reliability

Cronbach’s alpha was 0.93 for the total TEAM-NL scale and 0.90 for the seven-item Teamwork subscale. For the two-item Leadership and Task Management subscales, the inter-item correlations were 0.74 and 0.66, with Spearman-Brown coefficients of 0.85 and 0.80, respectively. Inter-item correlations for the total scale ranged from 0.45 to 0.74 (mean 0.56); the mean inter-item correlation for the Teamwork subscale was 0.57. Corrected item-total correlations ranged from 0.66 to 0.75 (Table 2), and deletion of any item did not increase alpha. Sensitivity analyses yielded comparable internal consistency across scenarios and rating positions (Cronbach’s alpha 0.87–0.94; Supplement 4).

**Table 2:** Item-level psychometric properties of the TEAM-NL (*n* = 299 instructor ratings).

	**Corrected item-total correlation**^a^	**Missing** ***n* (%)**	**Floor**^b^ ***n* (%)**	**Ceiling**^b^**, *n* (%)**		
				**Total**	**Scenario**^c^**1**	**Scenario**^c^**2**
Total score	−	3 (1.0)	0 (0.0)	8 (2.7)	2 (1.3)	6 (4.1)
Item 1	0.71	0 (0.0)	0 (0.0)	69 (23.1)	23 (15.4)	46 (30.7)
Item 2	0.75	5 (1.7)	3 (1.0)	86 (28.8)	28 (18.8)	58 (38.7)
Item 3	0.74	0 (0.0)	0 (0.0)	58 (19.4)	12 (8.1)	46 (30.7)
Item 4	0.73	3 (1.0)	0 (0.0)	70 (23.4)	21 (14.1)	49 (32.7)
Item 5	0.74	1 (0.3)	0 (0.0)	64 (21.4)	22 (14.8)	42 (28.0)
Item 6	0.70	5 (1.7)	0 (0.0)	108 (36.1)	38 (25.5)	70 (46.7)
Item 7	0.73	3 (1.0)	0 (0.0)	67 (22.4)	23 (15.4)	44 (29.3)
Item 8	0.73	8 (2.7)	0 (0.0)	68 (22.7)	26 (17.4)	42 (28.0)
Item 9	0.66	1 (0.3)	0 (0.0)	67 (22.4)	22 (14.8)	45 (30.0)
Item 10	0.72	0 (0.0)	1 (0.3)	59 (19.7)	19 (12.8)	40 (26.7)
Item 11	0.70	3 (1.0)	0 (0.0)	61 (20.4)	25 (16.8)	36 (24.0)
Item 12	−	2 (0.7)	0 (0.0)	5 (1.7)	2 (1.3)	3 (2.0)

a Based on forms with complete data for items 1–11 (*n* = 275; listwise deletion).

b Floor and ceiling effects were defined as the proportion of ratings at the lowest or highest possible score: 0 and 4 for items 1–11, 1 and 10 for item 12 and 0 and 44 for the total score. Proportions >15% were considered to indicate a floor or ceiling effect.

c Percentages for scenario 1 and scenario 2 are based on 149 and 150 instructor ratings, respectively (total score: 149 and 147 ratings with a valid total score).

Inter-rater reliability for two instructors was ICC(1,2) = 0.68 (95% CI 0.53–0.79, *p* < 0.001; *n* = 100). For performances with three valid scores (*n* = 88), ICC(1,3) was 0.78 (95% CI 0.69–0.85, *p* < 0.001). To assess the influence of repeated performances within participant groups, inter-rater reliability was also estimated separately for each scenario, so that each group contributed one performance to each analysis. ICC(1,2) was 0.64 (95% CI 0.36–0.79, *p* < 0.001; *n* = 50) in scenario 1 and 0.62 (95% CI 0.34–0.79, *p* < 0.001; *n* = 50) in scenario 2. For three raters, ICC(1,3) was 0.74 (95% CI 0.57–0.85, *p* < 0.001; *n* = 46) and 0.77 (95% CI 0.62–0.87, *p* < 0.001; *n* = 42), respectively.

### Acceptability and score distribution

Missing data per item ranged from 0% to 2.7%. Overall, 273 of 299 forms (91.3%) were complete for all 12 items and 275 (92.0%) for items 1–11. No floor effects were observed (≤1% of ratings at the minimum score for each item). Ceiling effects were observed for all items, ranging from 19.4% to 36.1%, and were higher in scenario 2 than in scenario 1 for all items (Table 2). At the total score level, 2.7% of ratings were at the maximum score.

### Validity

The TEAM-NL total score correlated strongly with the global performance rating (*r* = 0.86, *ρ* = 0.85, *p* < 0.001; Fig. 2, Table 3). Correlations between the global rating and the subscales ranged from 0.75 (Leadership and Task Management) to 0.82 (Teamwork), supporting convergent validity.

**Fig. 2:**
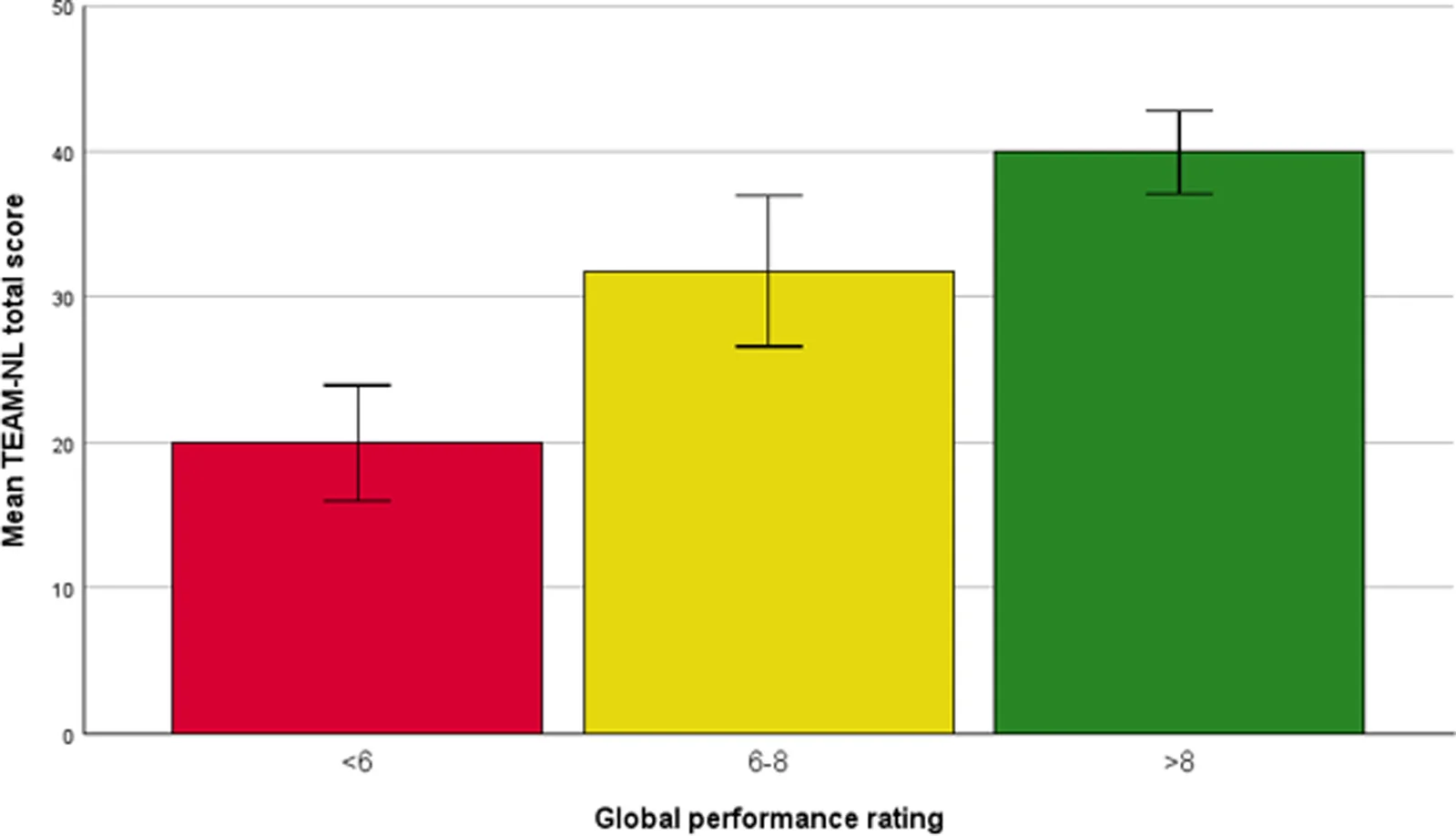
**Mean TEAM-NL total score by global performance rating category**. Error bars represent ±1SD. Total scores were prorated for forms with one missing item.

**Table 3:** Psychometric properties of the TEAM-NL compared with the original TEAM.

	**TEAM-NL**	**Original TEAM**^20^
Setting and sample	Live rating of 104 simulated team performances; 299 ratings by ALS instructors	56 video-recorded events rated by one expert^a^; 15 simulated events in real time^b^
Cronbach’s *α*	0.93^d^	0.89^b^
Inter-item correlation	*r* = 0.45–0.74^d^	*ρ* = 0.62–1.00^a^
Item-total correlation	*r* = 0.66–0.75 (corrected)^d^	*ρ* = 0.80–0.94 (correction not reported)^a^
Inter-rater reliability	ICC(1,2)^c^ = 0.68 (95% CI 0.53–0.79)	Mean item-level ICC = 0.60 *κ* = 0.55 (6 events, 2 raters)
Correlation total score-global rating	*r* = 0.86; *ρ* = 0.85	*ρ* = 0.95^a^

*Abbreviations:* ALS, Advanced Life Support; ICC, intraclass correlation coefficient; *κ*, Cohen’s kappa; *r*, Pearson’s correlation coefficient; *ρ*, Spearman’s rank correlation coefficient.

a 56 video-recorded events (3 hospital; 53 simulated), rated by one expert.

b 15 simulated events (three teams) rated in real time, each team by one of three instructors.

c ICC of the total score; not directly comparable with the mean of item-level ICCs reported for the original TEAM.

d Based on forms with complete data for items 1–11 (*n* = 275).

At the participant-group level, TEAM-NL scores were higher in scenario 2 (mean 34.4, SD 5.4) than in scenario 1 (mean 30.1, SD 5.6), *t*(51) = 4.14, *p* < 0.001. The mean difference was 4.3 points (95% CI 2.2–6.4), corresponding to a medium effect size (Cohen’s *d_z_* = 0.57, 95% CI 0.28–0.87).

A complete-case sensitivity analysis yielded comparable results (ICC(1,2) = 0.67, 95% CI 0.50–0.79; ICC(1,3) = 0.78, 95% CI 0.67–0.85; mean difference between scenarios 4.3 points, 95% CI 2.2–6.5).

## Discussion

This study demonstrates that the Dutch version of the Team Emergency Assessment Measure (TEAM-NL) has high internal consistency, convergent validity and acceptability for instructor-based assessment of team performance in simulation-based resuscitation training. Inter-rater reliability was moderate when two instructors’ ratings were averaged and good when three were averaged. Overall, the findings support the use of the TEAM-NL for structured instructor-based evaluation in Dutch interprofessional resuscitation training.

The high internal consistency observed in this study aligns with the original TEAM validation and previous language adaptations.^20^, ^22^, ^24^, ^25^, ^26^ The increase in inter-rater reliability from two to three raters is consistent with the expected gain in score reliability when ratings from multiple observers are aggregated.^34^, ^35^, ^36^ Variability between raters reflects the interpretative nature of performance assessment, in which observers evaluate complex and dynamic behaviours such as leadership, communication, and situation awareness. Differences in professional background, clinical experience, and attentional focus may lead raters to prioritize different aspects of team performance.^37^, ^38^ In line with generalizability theory, aggregating ratings across raters reduces random error and improves score stability.^39^

The absence of formal rater training may have contributed to the moderate reliability for two raters. Structured rater training is recommended to reduce rater error in simulation-based assessment, and high inter-rater agreement has been reported after such training.^40^, ^41^ Two other factors are likely to have contributed. First, ICCs depend on between-subject variance so that low heterogeneity among the rated teams lowers ICCs even when raters agree.^33^ The relatively homogeneous population of experienced ALS participants and the observed ceiling effects restricted score variability, whereas higher ICCs were reported in a study that deliberately included teams of differing expertise.^22^ Second, instructors rated performances in real time from different positions in the room, whereas several previous studies used video recordings, which ensure that all raters observe the same events.^22^, ^25^ Under these conditions, averaging the ratings of three instructors nevertheless provided good reliability. In practice, ratings by one or two instructors may suffice for formative feedback, whereas averaging across three instructors is preferable when scores are used for assessment or research.

The dataset had a hierarchical structure, with multiple instructor ratings nested within observed team performances and two performances contributed by each participant group. Internal consistency remained high in scenario- and rating-position-specific analyses, and scenario-specific ICC estimates were comparable with the primary estimates, as were the results of the complete-case analysis. These results suggest that the reliability estimates were robust to the dependency structure and to the handling of missing data.

Convergent validity was supported by the strong association between the TEAM-NL total score and global performance rating. This correlation was somewhat lower than in the original study,^20^ in which a single expert rated video-recorded events; ratings by multiple instructors in a live setting are likely to introduce more variability. Because the total score and the global rating were provided by the same rater at the same moment, shared method variance may have inflated this association.

TEAM-NL scores were higher in the second scenario. Although this finding is consistent with responsiveness to performance change during training, differences in scenario content and in the instructors rating each scenario preclude attributing the change exclusively to learning. The higher ceiling effects in the second scenario may partly reflect performance improvement and increasing team familiarity, although scenario content may also have contributed.^42^ Ceiling effects have been reported in other TEAM adaptations, particularly in experienced teams,^22^, ^24^ whereas scores of medical and nursing students in the original real-time testing clustered in the mid-range.^20^ This suggests that ceiling effects are related to the experience level of the population rather than to the translation. Items reflecting team morale and the leader’s global perspective showed the highest ceiling effects. Although ceiling effects may reduce discrimination among high-performing teams at item level, only 2.7% of total scores reached the maximum, indicating that the total score retained its capacity to differentiate between teams.

This study has several strengths. It followed a rigorous cross-cultural translation process and included a relatively large sample of observed performances, each rated by multiple instructors. Compared with the original validation, in which inter-rater reliability was examined on six video-recorded events using item-level statistics,^20^ reliability was estimated at the score level in a considerably larger sample of live-rated performances. Moreover, the pragmatic design, with certified ALS instructors rating in authentic course settings without additional rater training, reflects routine educational practice and enhances the applicability of the findings.

Several limitations should be considered. First, the study was conducted in simulation-based training at two training centres, which may limit generalizability to clinical practice and other settings, and instructors received no specific training in the use of the TEAM-NL. Second, the two scenarios differed in content and were rated by different instructors without recording instructor identity; change in scores between scenarios can therefore not be attributed solely to learning, and rater effects could not be modelled. Third, convergent validity was based on a global rating by the same rater, which may have inflated the association, and structural validity was not examined. Future studies with larger samples could examine the factor structure of the TEAM-NL using multilevel confirmatory factor analysis, which accounts for the nesting of ratings within performances and participant groups. Finally, the number of performances available for ICC(1,2) was slightly below the planned 102, and the lower bound of its 95% CI fell below the predefined minimum of 0.60.

## Conclusion

The TEAM-NL tool showed high internal consistency, convergent validity and acceptability for instructor-based assessment of team performance during simulation-based resuscitation training. Inter-rater reliability was moderate for two raters and good when the ratings of three instructors were averaged. Future research should explore its use in debriefing and in clinical practice, as well as the added value of participants’ self-assessments alongside instructor ratings.

## AI declaration

During the preparation of this manuscript, the authors used ChatGPT (OpenAI) and Claude (Anthropic) to improve readability and language, as the authors are not native English speakers. All analyses were performed and verified by the authors in SPSS. The authors reviewed and edited all content and take full responsibility for the manuscript.

## CRediT authorship contribution statement

**Frederik Marynen:** Writing – original draft, Validation, Conceptualization. **Lars Mommers:** Writing – original draft, Validation, Formal analysis, Conceptualization. **Dennie Wulterkens:** Validation, Investigation. **Thom Bormans:** Investigation. **Bas C.T. van Bussel:** Methodology, Formal analysis. **Iwan C.C. van der Horst:** Writing – review & editing. **Daniëlle Verstegen:** Writing – review & editing. **Diana Dolmans:** Writing – review & editing. **Walther N.K.A. van Mook:** Writing – review & editing, Validation. **Simon Cooper:** Writing – review & editing, Validation.

## Funding

This research did not receive any specific grant from funding agencies in the public, commercial, or not-for-profit sectors.

## Declaration of competing interest

The authors declare that they have no known competing financial interests or personal relationships that could have appeared to influence the work reported in this paper.
