# Supplementary material for: Translation and validation of the TEAM-NL instrument for assessing interprofessional team performance in simulation-based resuscitation training
Source: Resusc Plus. 2026 Oct 20;32:101535. doi: 10.1016/j.resplu.2026.101535 (PMC13756183; doi:10.1016/j.resplu.2026.101535)
Supplement: Supplementary file 1 [file mmc1.docx]

**Supplement 1, Original *TEAM* author’s comments on the back-translation**

| **Backward translation** | **Original *TEAM* author’s comments** |
| --- | --- |
| Town / city | Place is not intended like this, more like a ward or department |
| 1. The team leader informed the team about what was expected of them through instructions and assignments | Is there a 'stronger' word meaning 'command'? |
| 2. The team leader kept a general overview. Prompts: Did he/she monitor clinical procedures and the surroundings? Did he/she stay neutral where applicable? Did he/she delegate tasks when necessary? | Not sure the word 'neutral' mirrors the original intent |
| 8. The team followed and reassessed the situation | OK, but is 'followed' actually the same as 'monitored'? |
| In general, | Don’t think 'general' is quite the same as 'overall' |

**Supplement 2, expert panel comments**

| **Before review** | **After review** |
| --- | --- |
| 7. Het team paste zich aan veranderende situaties aan.  Prompts: Aanpassing van de rol binnen hun beroepstaak? Bij veranderende situaties: Verslechtering van de patiënt? Aanpassingen in het team? | 7. Het team paste zich aan veranderende situaties aan.  *Prompts: Flexibiliteit binnen en tussen teamrollen? Open voor nieuwe ideeën?* |

**Supplement 3** **– Final TEAM-NL translation**

**Team Emergency Assessment Measure (TEAM)**

**Inleiding**

Deze vragenlijst over niet-technische vaardigheden is bedoeld als observationele score voor een valide, betrouwbare en haalbare beoordeling van medische spoedteams (bv. reanimatie- en traumateams). De vragenlijst moet worden ingevuld door een medisch deskundige om een nauwkeurige prestatiebeoordeling en feedback over leiderschap, teamprestaties, situatiebewustzijn en taakuitvoering mogelijk te maken. Waar van toepassing zijn hulpvragen (prompts) opgenomen. Voor elke beoordeling moet de volgende schaal worden gebruikt:

| Nooit / bijna nooit | Zelden | Ongeveer even vaak wel als niet | Vaak | Altijd / bijna altijd |
| --- | --- | --- | --- | --- |
| 0 | 1 | 2 | 3 | 4 |

**Teamidentificatie**

Datum:	Tijd:	Locatie:

Teamleider:	Teamleden:

**Leiderschap: aangenomen wordt dat de teamleider is aangewezen, zich heeft aangemeld, of de hoogste in rang was. Indien er geen teamleider was, geef dan score 0 bij vraag 1 en 2
 0	1	2	3	4**

1. **De teamleider informeerde het team over wat er van hen werd verwacht**

**middels instructies en opdrachten.**

**2. De teamleider behield een algemeen overzicht.**

*Prompts: Bewaakte de klinische procedures en hield toezicht op de omgeving?*

*Bleef 'hands off' waar van toepassing? Delegeerde taken indien nodig?*

**Teamprestatie: de beoordeling moet het team als geheel betreffen, 0	1	2	3	4**

**d.w.z. de teamleider en het team gezamenlijk (in meer of mindere mate).**

**3. Het team communiceerde effectief.**

*Prompts: Verbale, non-verbale en schriftelijke vormen van communicatie?*

**4. Het team werkte samen om de taken op tijd te voltooien.**

**5. Het team handelde doordacht en met kalmte.**

*Prompts: Emoties van toepassing? Problemen met conflictbeheersing?*

**6. Het moreel van het team was positief.**

*Prompts: Passende ondersteuning, vertrouwen, spirit, optimisme, besluitvaardigheid?*

**7. Het team paste zich aan veranderende situaties aan.**

*Prompts: Flexibiliteit binnen en tussen teamrollen? Open voor nieuwe ideeën?*

**8. Het team monitorde en herbeoordeelde de situatie.**

**9. Het team anticipeerde op mogelijke acties.**

*Prompts: Voorbereiding van defibrillator, medicijnen, beademingsapparatuur?*

**Taakuitvoering: 0	1	2	3	4**

**10. Het team prioriteerde taken.**

**11. Het team volgde goedgekeurde normen en richtlijnen.**

*Prompt: Was enige afwijking mogelijk gepast?*

**Algemeen: 1	2	3	4	5	6	7	8	9	10**

**12. Geef op een schaal van 1-10 uw algemene beoordeling**

**van de niet-technische prestaties van het team.**

**Comments:________________________________________________________________________**

**Supplement 4, Internal consistency of the TEAM-NL stratified by scenario and rating position**

| Scenario | Overall | Rating position 1 | Rating position 2 | Rating position 3 |
| --- | --- | --- | --- | --- |
| 1 | 0.93 | 0.93 | 0.93 | 0.93 |
| 2 | 0.93 | 0.87 | 0.94 | 0.93 |

Cronbach's α for the 11-item TEAM-NL scale, based on forms with complete data for items 1–11. Rating position reflects the data-entry order, which was arbitrary and not determined by instructor characteristics. N per cell: scenario 1, overall 139 (positions 1–3: 49, 47, 42), scenario 2, Overall 136 (46, 49, 39). Overall includes three ratings in fourth position, which are not shown separately.
